# Evaluation of the Properties, Gas Permeability, and Selectivity of Mixed Matrix Membrane Based on Polysulfone Polymer Matrix Incorporated with KIT-6 Silica

**DOI:** 10.3390/polym11111732

**Published:** 2019-10-23

**Authors:** Sie Hao Ding, Tiffany Yit Siew Ng, Thiam Leng Chew, Pei Ching Oh, Abdul Latif Ahmad, Chii-Dong Ho

**Affiliations:** 1 Department of Chemical Engineering, Universiti Teknologi PETRONAS, 32610 Seri Iskandar, Perak, Malaysia; ding.sie_g03642@utp.edu.my (S.H.D.); tiffany_16001691@utp.edu.my (T.Y.S.N.); peiching.oh@utp.edu.my (P.C.O.); 2 CO_2_ Research Centre (COSRES), Institute of Contaminant Management, Universiti Teknologi PETRONAS, 32610 Seri Iskandar, Perak, Malaysia; 3 School of Chemical Engineering, Engineering Campus, Universiti Sains Malaysia, 14300 Nibong Tebal, Pulau Pinang, Malaysia; chlatif@usm.my; 4 Department of Chemical and Materials Engineering, Tamkang University, New Taipei City 25137, Taiwan; cdho@mail.tku.edu.tw

**Keywords:** KIT-6, polysulfone, MMMs, CO_2_, gas permeability and selectivity

## Abstract

Mixed matrix membranes (MMMs) separation is a promising technology for gas permeation and separation involving carbon dioxide (CO_2_). However, finding a suitable type of filler for the formation of defect-free MMMs with enhancement in gas permeability remains a challenge. Current study focuses on synthesis of KIT-6 silica and followed by the incorporation of KIT-6 silica as filler into polysulfone (PSF) polymer matrix to fabricate MMMs, with filler loadings of 0–8 wt %. The effect of KIT-6 incorporation on the properties of the fabricated MMMs was evaluated via different characterization techniques. The MMMs were investigated for gas permeability and selectivity with pressure difference of 5 bar at 25 °C. KIT-6 with typical rock-like morphology was synthesized. Incorporation of 2 wt % of KIT-6 into PSF matrix produced MMMs with no void. When KIT-6 loadings in the MMMs were increased from 0 to 2 wt %, the CO_2_ permeability increased by ~48%, whereas the ideal CO_2_/CH_4_ selectivity remained almost constant. However, when the KIT-6 loading in PSF polymer matrix was more than 2 wt %, the formation of voids in the MMMs increased the CO_2_ permeability but sacrificed the ideal CO_2_/CH_4_ selectivity. In current study, KIT-6 was found to be potential filler for PSF matrix under controlled KIT-6 loading for gas permeation.

## 1. Introduction

Polymer membranes have been used industrially for gas separation. Their ease to scale-up, energy friendly, high efficiency, affordable, and simple operation make them economical favorable compared to conventional separation methods [1,2]. The common polymer membranes include cellulose acetate (CA), polyethersulfone (PESf), polyimide (PI), and polycarbonates (PC). Apart from these, polysulfone (PSF) is gaining great attention in recent years because PSF exhibits some important benefits such as good thermomechanical stability, high plasticization resistance (above 30 bar), and good gas permeability and selectivity [3,4,5,6,7,8].

In recent years, mixed matrix membranes (MMMs) emerged as alternative candidates for gas permeation and separation [9,10,11,12,13]. MMMs, comprising inorganic materials embedded in a polymer matrix, combine the ease of processability of polymers with high permeability or selectivity of inorganic porous fillers [14]. Furthermore, by incorporating inorganic fillers into polymer matrix, there is extremely huge possibility a membrane with higher gas separation performance relative to the bare polymeric membrane material can be established [15].

There is various type of inorganic materials have been used as fillers in polymer matrix, for instance, carbon nanotubes, zeolites, carbon molecular sieves, and metal–organic frameworks. Nevertheless, the production of defect-free MMMs is challenging because of the incompatibility in physical and chemical properties between the polymer and inorganic phase. Microvoid formation resulting from weak interaction between polymer and filler may cause membrane separation performance decreases significantly [14,15].

Ordered mesoporous silica materials, such as MCM-41 and MCM-48, are inorganic materials used as fillers in MMMs to enhance the gas separation performance of membranes. These mesoporous materials possess several advantages such as high CO_2_ adsorption, high specific surface area, high mechanical and thermal stability. The high porosity of these materials facilitates the gas diffusion during gas separation [14].

In contrast to MCM-41 and MCM-48, KIT-6 is another type of larger pore size (~6 nm) mesoporous silica with a three-dimensional interconnected cubic pore structure. KIT-6 has been proved to have high affinity for various gases with its two intertwined systems of mesoporous channels which can also be connected via irregular micropores in the walls [16]. The large pore size of KIT-6 enables the formation of intimate composites, resulting from the penetration of polymer chain into the mesopores. Besides, the gas permeability can be enhanced due to easier diffusibility of gases through the large pore.

A numbers of studies have investigated the preparation and gas permeation studies of MMMs incorporated with mesoporous silica as filler. The incorporation of different type of fillers had different effects on the properties and the gas permeability of the formed MMMs. Wu et al. [17] investigated the CO_2_ permeability of poly(ether-block-amide) incorporated with MCM-41. The CO_2_ permeability was enhanced by 102.3% at 20 wt % filler loading of the MMMs [17]. Kim and Marrand [18] reported that incorporation of 40 wt % of MCM-41 into PSF increased the CO_2_ permeability by 275% compared to pristine PSF. Jomekian et al. [19] prepared the MMMs of PSF incorporated with MCM-48 increased the CO_2_ permeability by ~193% compared to pristine PSF. These studies show that incorporation of mesoporous silica into polymer membranes could enhance the gas permeability of the membranes.

In the current project, KIT-6 was used as filler with the aim to improve the CO_2_ gas permeability of PSF polymer matrix. Different loadings of 0–8 wt % of KIT-6 silica were incorporated into PSF polymer matrix to fabricate MMMs. The synthesized KIT-6 was characterized using field-emission scanning microscopy (FESEM), X-ray diffraction (XRD), high-resolution transmission electron microscopy (HRTEM), and N_2_ adsorption–desorption techniques. The effects of KIT-6 loading on the properties of fabricated MMMs were evaluated by characterization on the MMMs via FESEM and thermal gravimetric analysis (TGA). The fabricated MMMs were tested for gas permeability and selectivity at pressure difference of 5 bar.

## 2. Materials and Methods 

### 2.1. Synthesis of KIT-6 Silica 

KIT-6 silica was synthesized with the procedure reported previously with some modifications [20]. Pluronic P123 was dissolved in distilled water and concentrated hydrochloric acid (HCl, 37%) at 35 °C. After P123 was completely dissolved, butanol (BuOH) was added and stirred for an hour, followed by the addition of tetraethyl orthosilicate (TEOS). The resultant mixture is composed of TEOS:P123:HCl:H_2_O:BuOH in a 1:0.017:4.948:188:1.31 mole ratio. After 24 h of stirring, the mixture was hydrothermally treated under static condition at 35 °C for 24 h. Next, the mixture was filtered and washed with deionized water and followed by drying at 100 °C overnight in an oven. After drying, the sample was calcined for 6 h at 550 °C. 

### 2.2. Fabrication of the Membranes

For pristine PSF membrane fabrication, 3.1235 g of PSF pellets (average MW ~35,000 by LS; average Mn ~16,000 by MO) were added to 10 mL of tetrahydrofuran (THF) and stirred for 18 h using a magnetic bar. After PSF pellets were completely dissolved, the dope solution was subjected to sonication to remove trapped bubbles for 30 min. Then, the dope solution was casted on a glass plate by using casting knife. The polymer on the glass plate was covered and left 3 days in room temperature to ensure complete solvent evaporation. Then, the membrane was peeled off and stored for future usage.

For MMM fabrication, various loadings (2, 4, 6, 8 wt %) of KIT-6 were added, respectively, to 10 mL of THF followed by 30 min of ultrasonication. Then, PSF pellets were added and dissolved into KIT-6 solution for 24 h using a magnetic bar. After mixing for 18 h, the dope solution was subjected to sonication to remove trapped bubbles for 30 min. Then, the dope solution was casted on a glass plate by using casting knife. The MMMs on the glass plate were covered and left 3 days in room temperature to ensure complete solvent evaporation. The MMMs were peeled off and stored for future usage. The fabricated MMMs were named as 2%-KIT-6/PSF, 4%-KIT-6/PSF, 6%-KIT-6/PSF, and 8%-KIT-6/PSF for MMMs incorporated with KIT-6 loadings of 2, 4, 6, and 8 wt %, respectively.

### 2.3. KIT-6 Characterization

KIT-6 was subjected to X-ray diffraction (XRD, X’Pert^3^ Powder & Empyrean, PANalytical) scanning from 0.8 ° to 6 ° (2 theta) for crystalline structure study. The pore structure of KIT-6 was observed using high-resolution transmission electron microscopy (HRTEM, FEI Tecnai 20). The surface morphology of KIT-6 was revealed by using field-emission scanning electron microscope (FESEM, Zeiss Supra55 VP). N_2_ adsorption–desorption studies (TriStar II 3020) using liquid at nitrogen temperature of 77 K was used to study the pore characteristic of KIT-6. The specific surface area of KIT-6 was determined by using the Brunauer–Emmett–Teller (BET) technique. The mesopore pore size distribution was determined by using the Barrett–Joyner–Halenda (BJH) technique. 

### 2.4. Membranes Characterization 

The morphologies of the prepared membranes were analyzed by FESEM. Liquid nitrogen was used to break the pristine PSF membrane and KIT-6/PSF membranes to prepare a cross section of the membrane for FESEM analysis. Thermal gravimetric analysis (TGA, Perkin Elmer, STA-6000) with heating rate of 10 °C/min in nitrogen gas was used to study the weight change of the membranes with increasing temperature.

### 2.5. Gas Permeability and Selectivity Study

The gas permeability and selectivity study was carried out by feeding single gas CO_2_ gas and CH_4_ with 99.99% purity to the membrane. The membrane was sealed in a stainless steel permeation cell with permeate pressure of 1 bar. The feed pressure was regulated so that the pressure difference was 5 bar. The permeate flow was measured using bubble flow meter. The gas permeability was calculated using Equation (1).

(1) $$P=\;\frac{Nl}{\left(P_{f}-P_{p}\right)\;A}$$

where *P* is gas permeability across the membrane (Barrer, 1 × 10^−10^ cm^3^ (STP) cm/[cm^2^ s cmHg]), *N* is the permeate flow (cm^3^ s^−1^), *l* is membrane thickness (cm), *A* is the membrane area (cm^2^), *P*_f_ is the feed pressure (cm Hg), and *P*_p_ is the permeate pressure (cmHg).

The ideal selectivity, α*^i^*, of the membrane was calculated using Equation (2). 

(2) $$\alpha^{i}{}_{CO_{2}/CH_{4}}=P_{CO_{2}}/P_{CH_{4}}\;$$

## 3. Results and Discussion

### 3.1. Characterization of KIT-6

**Figure 1:**
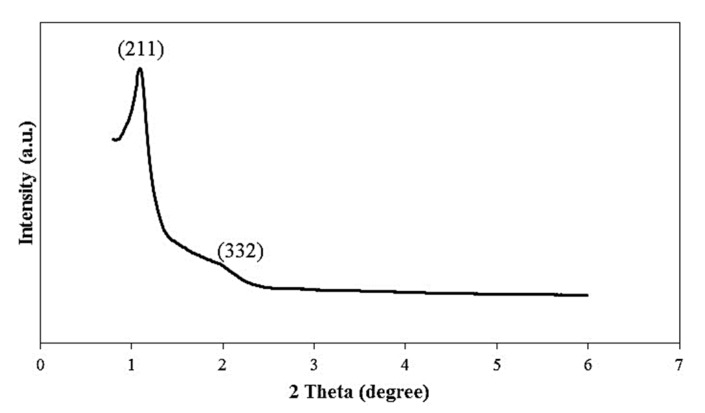
XRD pattern of KIT-6.

The powder XRD pattern of KIT-6 is shown in Figure 1. The peak at 1.09° shows that the sample possesses ordered mesostructure with a three-dimensional cubic Ia3d symmetry [16,21]. The XRD patterns represent two reflections, which can be assigned to (211) and (332). The XRD pattern of the KIT-6 sample synthesized in the current project agreed with the XRD pattern of KIT-6 reported by Ayad et al. [21].

**Figure 2:**
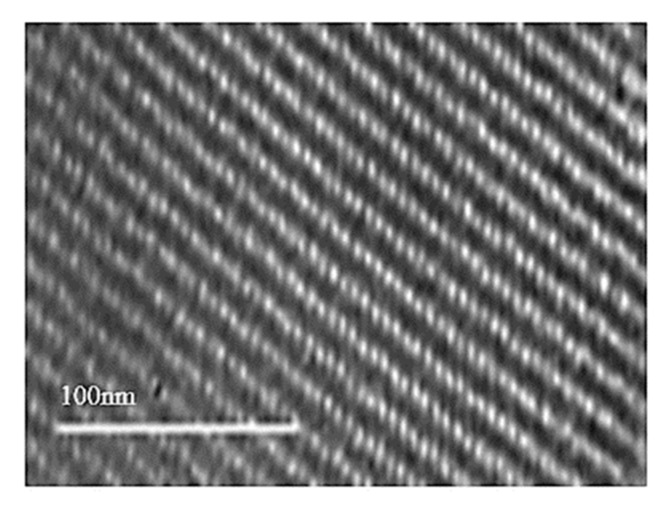
HRTEM image of KIT-6.

**Figure 3:**
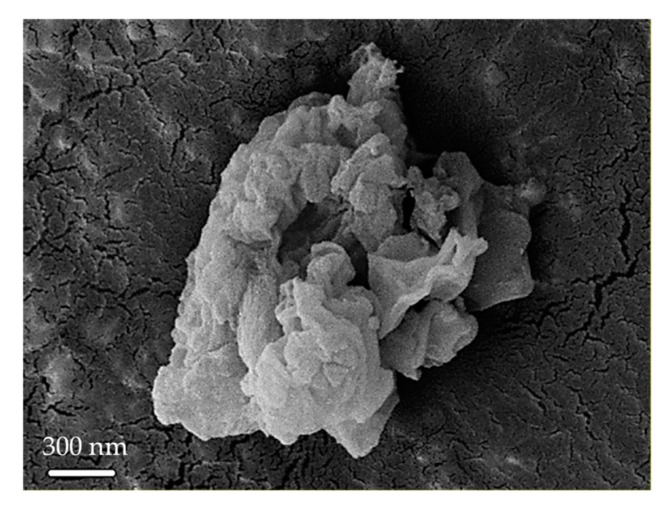
Field-emission scanning electron microscopy (FESEM) image of KIT-6.

Figure 2 shows the HRTEM images of synthesized KIT-6. The gyroidal cubic Ia3d structure of KIT-6 is observed in the HRTEM image in Figure 2 [22]. Figure 3 shows the FESEM image of the KIT-6. The FESEM image shows that the morphology of KIT-6 is typical rock-like morphology [14,15,23].

**Figure 4:**
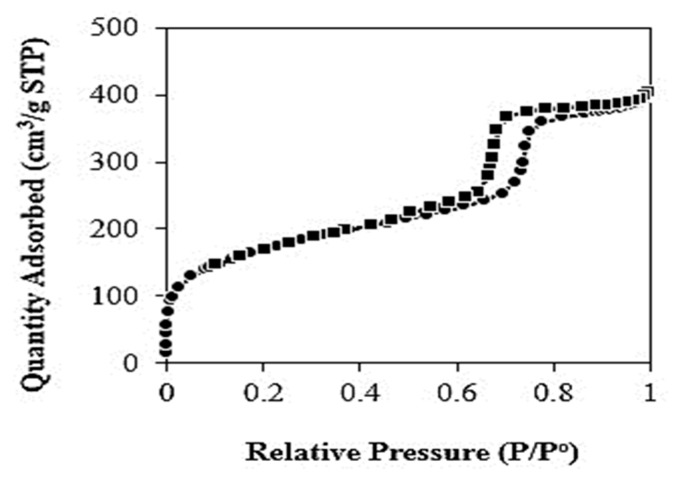
Nitrogen adsorption–desorption isotherm of KIT-6.

The nitrogen adsorption–desorption isotherm of KIT-6 is shown in Figure 4. From the nitrogen adsorption isotherms of KIT-6, type IV with a hysteresis loop can be observed which proved the characteristic of a mesoporous material [15,16,19,22,24]. The specific surface area, pore volume, and pore diameter of KIT-6 were determined to be 585 m²/g, 0.42 cm³/g, and 7.01 nm, respectively, which agreed with the data reported by Kishor & Ghoshal [22].

### 3.2. Membrane Characterization

Table 1 shows the weight loss percentage of the membranes via TGA analysis. Generally, weight loss occurred in 3 stages. In the first stage, desorption of physically absorbed water occurred at 30–500 °C. The main thermal degradation occurred from 500 to 580 °C. Most of the weight loss in the membranes occurred in this stage. Finally, at 580–800 °C, the samples were decomposed into ash. For pristine PSF membrane, the substantial weight loss started at around 500 °C which agreed with the onset temperature reported by other researchers [15,25,26]. MMMs with KIT-6 fillers also showed similar decomposition temperature with no obvious changes. 

**Figure 5:**
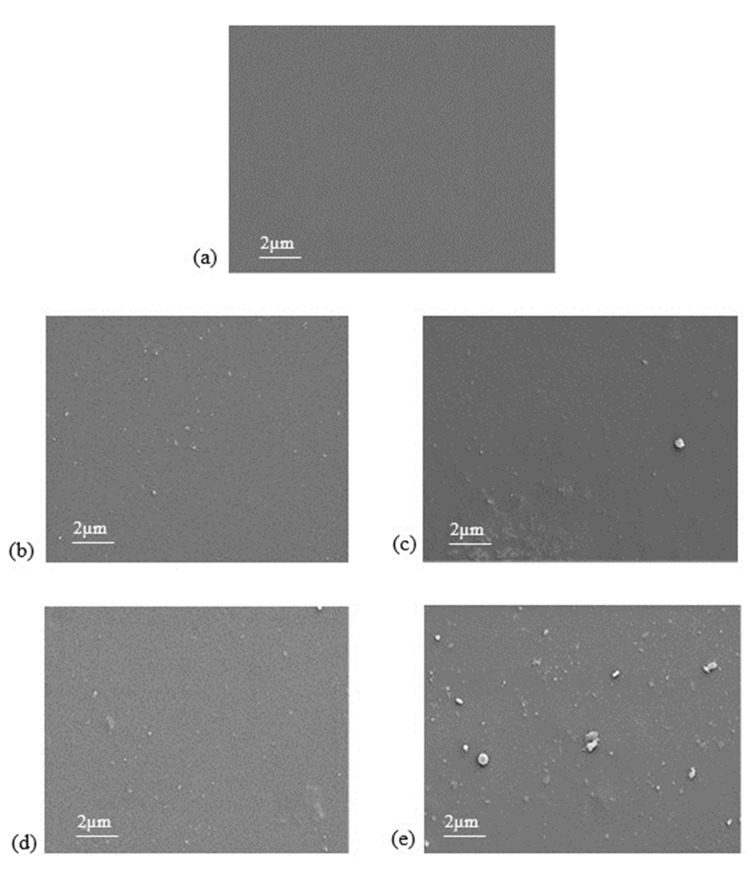
The top view FESEM images of (**a**) pristine polysulfone (PSF) membrane; (**b**) 2%-KIT-6/PSF; (**c**) 4%-KIT-6/PSF; (**d**) 6%-KIT-6/PSF; and (**e**) 8%-KIT-6/PSF.

Figure 5 shows FESEM images of surface morphology for pristine PSF membrane and MMMs. Smooth and clear surface morphology is observed for pristine PSF membrane in Figure 5a. It is observed that serious agglomeration of KIT-6 is observed in the MMMs when the KIT-6 loading was at 8 wt %.

**Figure 6:**
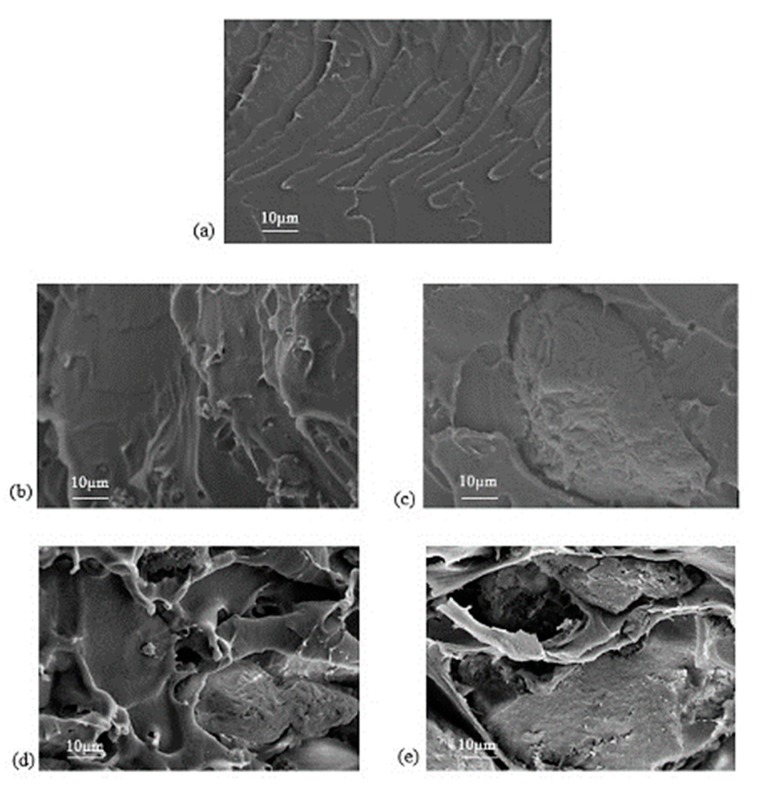
The cross-sectional images of (**a**) pristine PSF membrane; (**b**) 2%-KIT-6/PSF; (**c**) 4%-KIT-6/PSF; (**d**) 6%-KIT-6/PSF; and (**e**) 8%-KIT-6/PSF.

Figure 6 shows FESEM images of the cross-sectional morphology of pristine PSF membrane and MMMs. A dense structure can be seen in the pristine PSF membrane as shown in Figure 6a. No void was found for MMMs when 2 wt % of KIT-6 were incorporated into PSF matrix. However, serious void formation was observed in the MMMs when the KIT-6 loading was more than 4 wt %. This might be due to the occurrence of KIT-6 agglomeration at higher loading, which disturbed the PSF polymer matrix. The hydroxyl group attached on surface of silica caused KIT-6 tend to agglomerate with each other easily via hydrogen bonding [14,18,19].

### 3.3. CO_2_ Permeability and Selectivity of the Membranes

**Figure 7:**
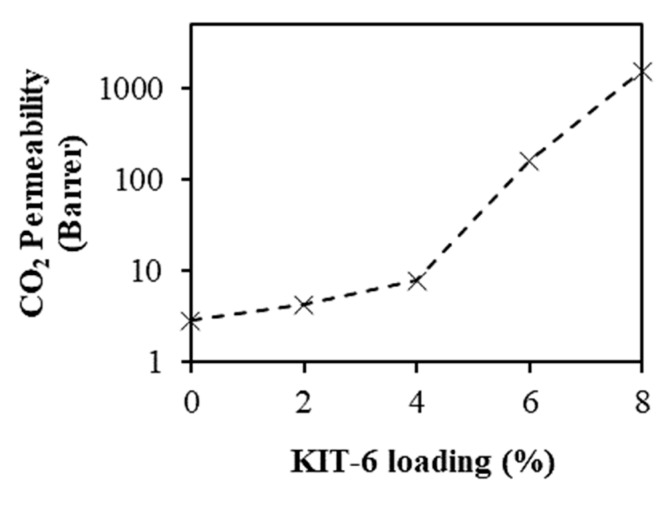
Effect of KIT-6 loading on CO_2_ permeability at 5 bar pressure difference and 25 °C.

**Figure 8:**
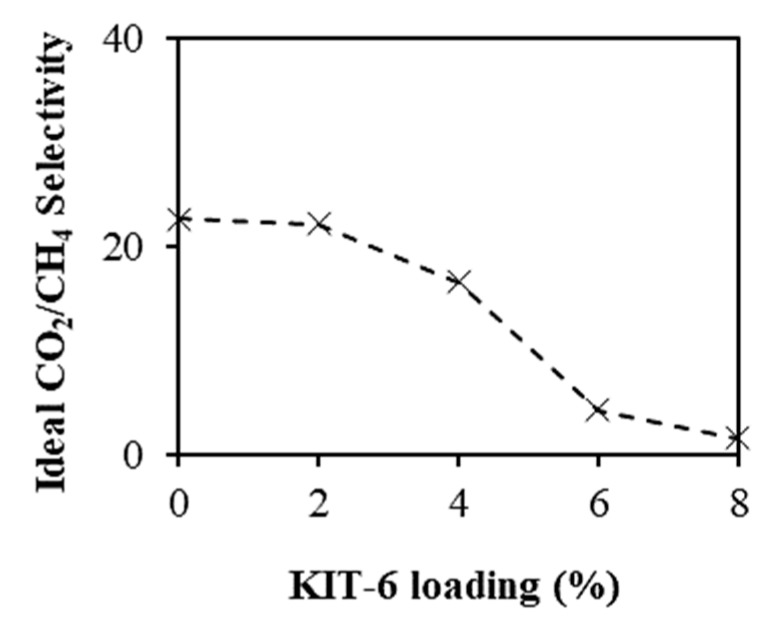
Effect of KIT-6 loading on CO_2_/CH selectivity at 5 bar pressure difference and 25 °C.

Figure 7 shows the CO_2_ permeability of the membranes, whereas Figure 8 shows the ideal CO_2_/CH_4_ selectivity at different KIT-6 loadings. When KIT-6 loadings in the MMMs were increased from 0 to 2 wt %, the CO_2_ permeability increased by ~48%, whereas the ideal CO_2_/CH_4_ selectivity is observed to be almost constant. The increase of CO_2_ diffusivity was due to preferable permeation of CO_2_ through the three-dimensional mesopores of KIT-6 loaded into the MMMs and the disruption of polymer chain packing in the presence of KIT-6 filler [16]. When the KIT-6 loading was increased to 2 wt %, more CO_2_ was able to permeate through the mesopores of KIT-6 in the MMMs [14,27]. On the other way, the CO_2_ permeability increased, but the ideal CO_2_/CH_4_ selectivity decreased when KIT-6 loading was more than 2 wt %, which could be due to the voids formation in the MMMs. The void formation became more serious when the KIT-6 loading was increased to beyond 4 wt %, as observed from FESEM images of cross-sectional morphology in Figure 6d,e [28]. The formed voids between the KIT-6 and PSF matrix might create bypassing channels for the permeation of the gas molecules. Consequently, the gas molecules tend to pass through the bypassing channels created by the voids in the MMMs with less resistance instead of passing through the mesoporous pore channel of KIT-6. This leaky interface caused increase in the gases permeability of membrane but sacrificing the ideal CO_2_/CH_4_ selectivity when the KIT-6 loading was more than 2 wt % [29].

Several researches have reported on the usage of MMMs incorporated by mesoporous silica for CO_2_ gas permeation. Waheed et al. [27] reported that the CO_2_ permeability increased ~6% when the PSF membrane was incorporated with 10 wt % rice husk silica (RHS) filler. Besides, the increment in CO_2_ permeability of ~89% was also reported by Kim and Marrand [30] when 10 wt % of MCM-48 was incorporated into the PSF polymer. Khan et al. [28] also reported that CO_2_ permeability was increased ~16% when Matrimid polymer was incorporated with 10 wt % of MCM-41 silica. On the other hand, Khan et al. [31] presented that the CO_2_ permeability raised for about 21% by incorporating 10 wt % of COK-12 into Matrimid polymer.

Research works have also reported on CO_2_ gas permeation using MMMs incorporated by fillers, which are other than mesoporous silica. Pakizeh et al. [32] reported increment of CO_2_ permeability of ~8% when 20 wt % 4A was incorporated into PSF polymer. On the other hand, Feijani et al. [33] incorporated MIL-53 into poly(vinylidene fluoride) based MMMs and CO_2_ permeability increased by ~32% when 5% of MIL-53 was incorporated into the MMMs. In another research work reported by Perez et al. [34], an approximately 28% increase in CO_2_ permeability was achieved when 23 wt % of MOP-18 was incorporated into Matrimid-based MMMs. In the current study, the ~48% increase in CO_2_ permeability, obtained by incorporating 2 wt % of KIT-6 into the MMMs, appears to be higher compared with the increase in CO_2_ permeability reported in many of the above-mentioned studies.

## 4. Conclusions

KIT-6 with typical rock-like morphology was synthesized and incorporated into the PSF matrix to form MMMs. The occurrence of serious agglomeration of KIT-6 in the MMMs is observed at KIT-6 loading of 8 wt %. Incorporation of 2 wt % of KIT-6 into PSF matrix produced MMMs with no void. When KIT-6 loadings in the MMMs were increased from 0 to 2 wt %, the CO_2_ permeability increased by ~48%, whereas the ideal CO_2_/CH_4_ selectivity remained almost constant. When KIT-6 loading was increased up to 2 wt %, the increase in CO_2_ permeability was due to preferable permeation of CO_2_ through the three-dimensional mesopores of KIT-6 loaded into the MMMs and the disruption of polymer chain packing in the presence of KIT-6 filler. Therefore, current study indicates that KIT-6 is potential filler for MMMs fabrication under controlled KIT-6 loading in order to increase the CO_2_ gas permeability performance of the membranes.

## Figures and Tables

**Table 1:** Weight loss percentage of membranes via TGA analysis [15,25,26].

Membrane Samples	Weight Percentage Loss (%)			
	Desorption of Absorbed Water at 30–500 °C	Main Thermal Degradation at 500–580 °C	Decomposition into Ash at 580–800 °C	Total Weight Loss
Pristine PSF	2.5	54.5	15.8	72.8
2%-KIT-6/PSF	4.4	64.2	13.5	82.1
4%-KIT-6/PSF	4.0	55.2	12.2	71.4
6%-KIT-6/PSF	1.7	54.3	10.0	66.0
8%-KIT-6/PSF	1.9	48.9	11.8	62.6
